# Complicated Diverticulitis in a 35-Year-Old Patient With Williams Syndrome: A Case Report

**DOI:** 10.7759/cureus.26604

**Published:** 2022-07-06

**Authors:** McKenzie M Raber, Sean M Bowling, Matthew Dorn

**Affiliations:** 1 Department of Surgery, Edward Via College of Osteopathic Medicine, Blacksburg, USA; 2 General Surgery, Johnston Memorial Hospital, Abingdon, USA

**Keywords:** preventative care, constipation, sigmoid diverticulitis, hartmann procedure, williams-beuren syndrome

## Abstract

Williams syndrome is caused by a deletion of the elastin gene on chromosome 7. One of the main roles of this gene is to maintain the strength and elasticity of the intestinal wall, and the absence of the elastin gene may predispose these patients to gastrointestinal pathology such as diverticulitis. Our patient was a 35-year-old Caucasian female with Williams syndrome who presented to the emergency department with diffuse abdominal pain for two days. A computed tomography (CT) scan of her abdomen and pelvis initially showed locally perforated sigmoid diverticulitis with pelvic abscess and acute peritonitis. Surgical management was indicated after the patient failed to respond to conservative treatment. She was treated with Hartmann’s procedure which showed purulent peritoneal fluid intraoperatively. Her hospital course was complicated by postoperative ileus and a peri-incisional abscess. After a 15-day hospital stay, she was discharged home with plans for ostomy reversal in six months. Patients with Williams syndrome have an increased risk of developing diverticulitis at a younger age than the general population due to their propensity for chronic constipation stemming from their child-like eating habits and low dietary fiber. Thus, we emphasize the importance of treating constipation in patients with Williams syndrome to prevent diverticulitis. If these patients present to the emergency department with acute diverticulitis, aggressive surgical management may be beneficial because rapid progression could ensue.

## Introduction

Williams syndrome is a genetic disorder caused by a sporadic microdeletion of the long arm of chromosome 7, which includes the elastin gene [1]. This deletion results in a wide range of clinical features such as elfin facial features, extreme friendliness, supravalvular aortic stenosis, and intellectual disabilities [2]. The elastin gene is responsible for maintaining connective tissue structure and integrity through the expression of tropoelastin [3]. Deletion of this gene is associated with the development of gastrointestinal pathology such as diverticulitis [1]. Diverticular disease is prevalent in as many as one-third of patients with Williams syndrome [4]. Further investigation showed that 8% of patients diagnosed with Williams syndrome may develop their first case of diverticulitis before the age of 40 compared to 2% in the general population in the same age cohort [5]. The youngest reported case was nine years old [6]. This patient group is also at higher risk for complications such as perforation or abscess formation [5]. This case report presents a 35-year-old female with Williams syndrome who presented to the emergency department with a perforated sigmoid colon secondary to diverticulitis.

## Case presentation

A 35-year-old Caucasian female with Williams syndrome, celiac disease, and mitral valve prolapse presented to the emergency department with diffuse abdominal pain for two days. At the time of surgical consultation, the patient was experiencing significant pain with movement that was relieved by lying still, worsening chronic constipation, but no fever, nausea, vomiting, or diarrhea. She was well-appearing with an extremely friendly, cheerful affect despite her pain. Vitals showed tachycardia but were otherwise normal. There was diffuse tenderness to palpation throughout the abdomen without guarding or rebound, and her bowel sounds were significantly decreased.

**Figure 1:**
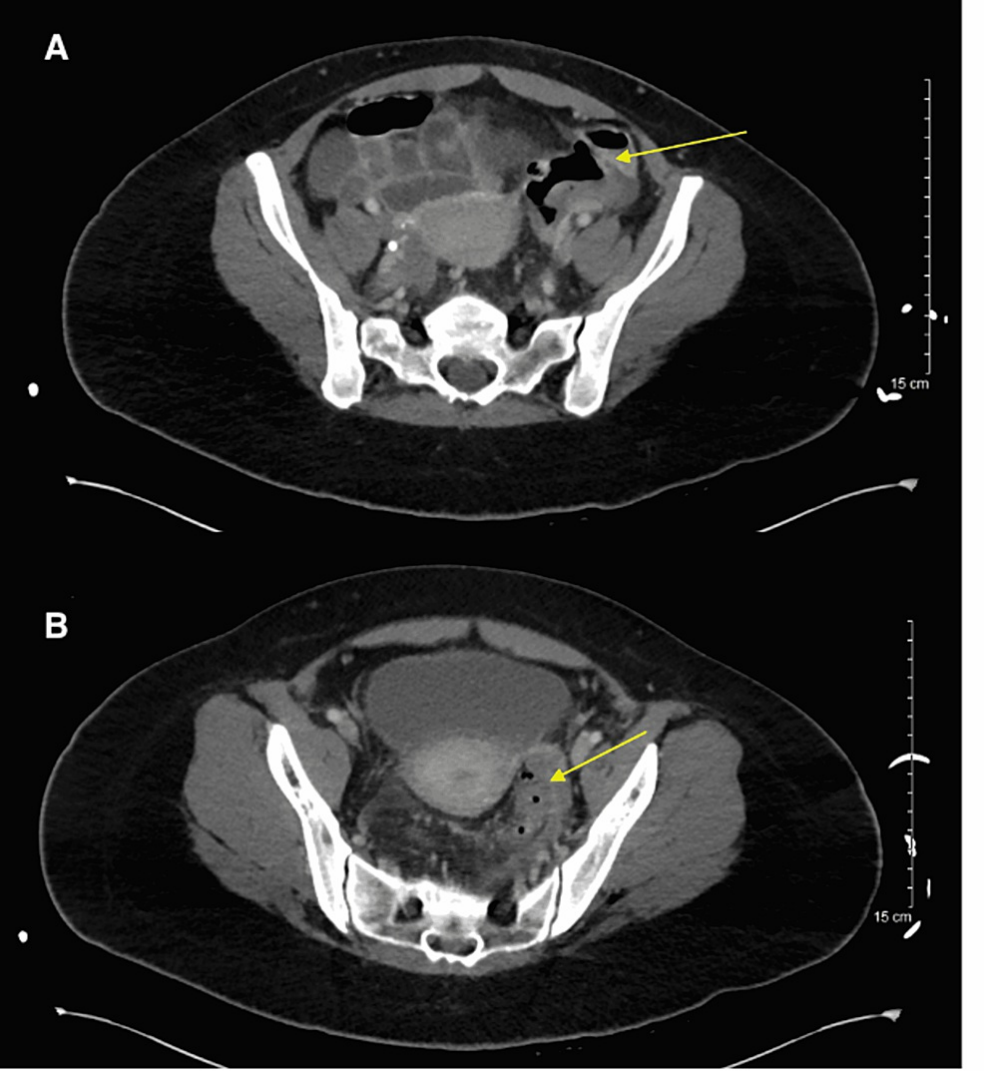
(A) Inflamed sigmoid colon with a pocket of free air adjacent (yellow arrow). (B) Inflamed colon with fluid and pockets of free air consistent with abscess formation (yellow arrow).

Her labs showed an elevated white blood cell count of 23.7 k/µL. A computed tomography (CT) scan of her abdomen and pelvis showed acute diverticulitis in the sigmoid colon with a small extraluminal air collection between the sigmoid colon and left lateral pelvic sidewall consistent with localized perforation (Figures 1A, 1B). She was diagnosed with perforated sigmoid diverticulitis with pelvic abscess, acute peritonitis, and sepsis without acute organ dysfunction. Given this diagnosis, she met the criteria for stage II of the modified Hinchey classification (Table 1) [7].

The initial treatment plan was conservative management with bowel rest, intravenous (IV) infusion of sodium chloride 0.9% at 125 mL/hour, 3.375 g of IV piperacillin/tazobactam every six hours, 4 mg IV morphine as needed, and 4 mg IV ondansetron as needed. Over the next nine hours, the patient complained of worsening pain and discomfort that could not be relieved with positional changes. After discussing treatment options with the patient and her caregivers in the setting of her worsening clinical condition, the patient elected for surgical treatment.

An exploratory laparotomy revealed perforated sigmoid diverticulitis with pelvic abscess and purulent fluid in the peritoneum. This intraoperative finding increased the severity of her condition to the modified Hinchey classification stage III (Table 1) [7]. A sigmoid colectomy with colostomy placement was performed and the incision was closed at the time of surgery. The culture of the purulent peritoneal fluid resulted in the growth of *Escherichia coli* and *Pseudomonas aeruginosa*.

**Table 1:** Modified Hinchey classification.

Stage	Modified Hinchey classification
0	Clinically mild diverticulitis
1a	Confined pericolic or phlegmonous inflammation
1b	Confined abscess formation (<5 cm)
II	Pelvic, retroperitoneal, or distal intra-abdominal abscess
III	Generalized purulent peritonitis
IV	Generalized fecal peritonitis

**Figure 2:**
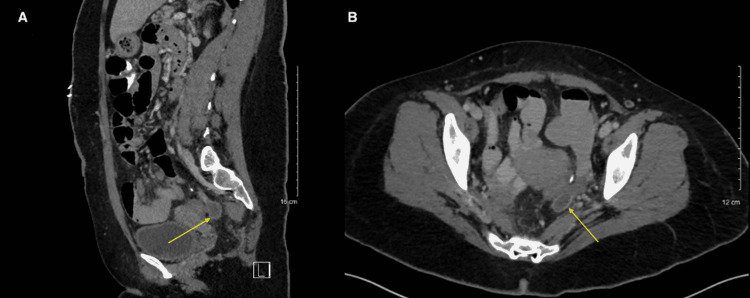
A small residual rim-enhancing fluid collection compatible with abscess measuring 2.2 × 2.0 × 1.0 cm is shown in A and B (yellow arrows).

The patient’s hospital stay was complicated by a postoperative ileus on postoperative day (POD) 2, which was resolved with bowel rest and IV fluids by POD 5. The patient also began producing liquid stool in the ostomy bag on POD 5. The patient continued to progress, and the Jackson-Pratt drain was removed on POD 9. On POD 10, purulent fluid was draining from the distal portion of the midline incision, and the leukocyte count was notably elevated at 13.4 k/µL from 11.8 k/µL on POD 9, so IV piperacillin/tazobactam was continued. The leukocyte count continued to rise to 16.1 k/µL on POD 12. An abdominal CT scan revealed a small, residual rim-enhancing fluid collection consistent with an abscess (Figures 2A, 2B). On POD 13, 1.75 g IV vancomycin every eight hours was started, and the white blood cell count dropped to 13.4 k/µL. On POD 15, the white blood cell count was within normal limits at 9.3 k/µL, and the patient was discharged with amoxicillin/clavulanate 300 mg twice daily for five days and levofloxacin 750 mg once daily for five days. The patient was prescribed home health for ostomy and wound care. She was seen in the clinic for follow-up one week later by which time she was healing well with no acute complaints.

## Discussion

While the surgical intervention provided in this case was routine, the case is unique in how the patient’s condition of Williams syndrome contributed to the presentation. Constipation, physical inactivity, and a low-fiber, high-fat diet are risk factors for the development of diverticulosis. In patients with Williams syndrome, diverticulosis is significantly more prevalent as their child-like eating habits consisting of foods high in carbohydrates and refined sugars with few vegetables make them more prone to constipation. Additionally, the elastin gene maintains the elastic properties of colonic tissue, and its deletion causes diminished colonic recoil and increases the likelihood of colonic distension and diverticula formation as a result [8]. One study showed that these patients are three times more likely to develop diverticulitis over their lifetime compared to the general population [5].

Patients with Williams syndrome also have an increased risk of developing diverticulitis at a younger age [5]. Therefore, it is important for providers to aggressively treat constipation in patients with Williams syndrome. These patients should be taught from a young age to eat high-fiber foods. The child-like behavior expressed by Williams syndrome patients into adulthood may interfere with compliance with most lifestyle behavior modifications [1,8]. Treating constipation with an over-the-counter fiber supplement may yield better results if it can be administered by a caregiver. These patients should also be counseled on the importance of physical activity and adequate water intake to prevent constipation. Furthermore, these patients must not be lost to follow-up as they transition from pediatric to adult primary care providers [4]. Interventions to prevent constipation gain value as these patients age and should be closely monitored throughout their lifetime.

It has been shown that patients with Williams syndrome are more likely to develop a complicated form of diverticulitis, hence, conservative therapy may not always be the best option for these patients [5]. Due to initial imaging supporting modified Hinchey classification stage II, we opted for treatment with antibiotics and pain medication. However, she became extremely uncomfortable and intolerant to pain. It was difficult to accurately assess her clinical status due to her friendly behavior. Although CT scan findings supported continued conservative treatment, due to her worsening clinical status, we decided that the Hartmann procedure was the best option for our patient after a discussion with the patient and her caregivers. Purulent fluid in the peritoneal cavity and sigmoid perforation found intraoperatively indicate that this patient progressed from stage II to stage III of the Hinchey classification in less than 12 hours. Similar cases have been reported in the literature, and we suspect that this clinical scenario is common in this patient population, and therefore, early aggressive treatment may be warranted to prevent rapid progression and complications that may ensue [9].

## Conclusions

All members of the care team should counsel patients with Williams syndrome about the importance of maintaining adequate fiber intake to prevent diverticulosis, a medical condition for which this patient group is at increased risk. Patients with this condition may also require more aggressive surgical management compared to the general population when diagnosed with perforated diverticulitis. Their friendly demeanor makes it difficult to assess clinical status, and the absence of elastase within their colonic wall may complicate the healing process. Providers should be aware of the potential for rapid progression of diverticulitis in this patient population.

## References

[1] Waz WR, Lee TM (2021). Williams syndrome. UpToDate.

[2] Bacino CA (2021). Microdeletion syndromes (chromosomes 1 to 11). UpToDate.

[3] Matisoff AJ, Olivieri L, Schwartz JM, Deutsch N (2015). Risk assessment and anesthetic management of patients with Williams syndrome: a comprehensive review. Paediatr Anaesth.

[4] Pober BR (2010). Williams-Beuren syndrome. N Engl J Med.

[5] Partsch CJ, Siebert R, Caliebe A, Gosch A, Wessel A, Pankau R (2005). Sigmoid diverticulitis in patients with Williams-Beuren syndrome: relatively high prevalence and high complication rate in young adults with the syndrome. Am J Med Genet A.

[6] Ignacio RC Jr, Klapheke WP, Stephen T, Bond S (2012). Diverticulitis in a child with Williams syndrome: a case report and review of the literature. J Pediatr Surg.

[7] Tochigi T, Kosugi C, Shuto K, Mori M, Hirano A, Koda K (2018). Management of complicated diverticulitis of the colon. Ann Gastroenterol Surg.

[8] Pemberton JH (2021). Colonic diverticulosis and diverticular disease: epidemiology, risk factors, and pathogenesis. UpToDate.

[9] Deshpande AV, Oliver M, Yin M, Goh TH, Hutson JM (2005). Severe colonic diverticulitis in an adolescent with Williams syndrome. J Paediatr Child Health.

